# Complete Mitochondrial Genome of *Chlorogomphus papilio* (Odonata: Anisoptera: Chlorogomphidae) and Phylogenetic Analyses

**DOI:** 10.3390/biology14050493

**Published:** 2025-05-01

**Authors:** Xiaoxiao Jin, Xiaojia Lin, Simeng Wang, Jie Fang

**Affiliations:** 1 School of Life Sciences, Anhui University, Hefei 230601, China; 2 Technology Center of Hangzhou Customs District, Hangzhou 310016, China; lxj@zaiq.org.cn; 3 School of Resources and Environmental Engineering, Anhui University, Hefei 230601, China

**Keywords:** Chlorogomphidae, mitochondrial genome, phylogenetic tree, *Chlorogomphus papilio*

## Abstract

This study aimed to elucidate the mitochondrial genome organization of *Chlorogomphus papilio* and the phylogenetic relationships of Chlorogomphidae. We used the Illumina MiSeq sequencing platform to sequence the mitochondrial genome of *C. papilio*, which was subsequently assembled, annotated, and analyzed. Bayesian inference, maximum likelihood, and maximum parsimony methods were employed to construct the mitochondrial phylogenetic tree of 25 species of Chlorogomphidae based on *16S rRNA* and *cox1* genes. We observed that the mitochondrial genome of *C. papilio* is 15,251 bp in length and includes 13 protein-coding genes (PCGs), 22 tRNA genes, 2 rRNA genes, and a non-coding control region. All PCGs start with a typical ATN codon. While *cox1*, *cox2*, *cox3*, and *nad5* end with an incomplete termination codon (T), the remaining PCGs terminate with TAG. The secondary structure of the 22 tRNAs showed that only the *trnS1* gene lacked the dihydrouracil arm (DHU arm), whereas the rest formed a typical cloverleaf structure. Additionally, 32 G-U mismatches were observed in the secondary structure. Phylogenetic analyses indicated that *C. papilio* and *C. magnificus* are sister species. Divergence time analyses indicated that Chlorogomphidae originated around 111.04 Ma, with *C. papilio* diverging from the common ancestor shared with *C. magnificus* approximately 58.51 Ma. This divergence is likely linked to the Paleocene–Eocene Thermal Maximum (PETM) and the tectonic uplift of the Himalayas, which created warm, humid habitats and contributed to geographic isolation. This study contributes to a better understanding of the mitochondrial genome and phylogeny of *C. papilio*, providing valuable molecular markers for further genetic studies.

## Simple Summary

In this study, we sequenced and analyzed the mitochondrial genome of *Chlorogomphus papilio* (Ris, 1927). The genome was 15,251 bp in length and contained 13 protein-coding genes, 22 tRNA genes, two rRNA genes, and one non-coding region. The mitochondrial phylogenetic tree of Chlorogomphidae, constructed based on *16S rRNA* and *cox1* genes, indicated that *C. magnificus* and *C. papilio* are sister species. Divergence time analyses indicated that Chlorogomphidae originated around 111.04 Ma, with *C. papilio* diverging from the common ancestor shared with *C. magnificus* approximately 58.51 Ma, likely influenced by the Paleocene–Eocene Thermal Maximum and the tectonic uplift of the Himalayas. The data obtained from our study could serve as a valuable resource for future research on the evolution and conservation of *C. papilio*.

## 1. Introduction

*Chlorogomphus papilio* is an ancient species with incomplete metamorphosis, belonging to the family Chlorogomphidae within the order Odonata [1]. It was first discovered in Guangdong, China, by Ris in 1927 [2]. *C*. *papilio* exhibits a wingspan exceeding 150 mm in females, representing the largest wingspan recorded among dragonflies in China, and is capable of sustained long-distance flight. Males are known to patrol extensive areas, sometimes flying several kilometers through valleys, which makes them one of the most wide-ranging species within Chlorogomphidae. Species of *Chlorogomphus* are highly sensitive to environmental conditions, with their presence closely tied to water quality and riparian vegetation. As a result, they serve as effective bioindicators, reflecting the quality of headwater streams during the larval stage and the condition of forest vegetation during adulthood [3,4,5].

The classification and phylogenetic relationships of Chlorogomphidae remain controversial. Originally, Carle Frank Louis proposed that Chlorogomphidae was closely related to a branch of Libellulidae [6], while Ishida supported its classification as a subfamily of Cordulegastridae [7]. Subsequent molecular studies have revealed that Chlorogomphidae is a sister group of Cordulegastridae, which is a part of Cordulegasteroidae [8,9]. On this basis, phylogenetic relationships within the family Chlorogomphidae were further explored. *Chlorogomphus* was initially placed within Cordulegastridae [10] but was later classified under Chlorogomphidae [6]. Today, Chlorogomphidae is a small family consisting of three widely recognized genera: *Chlorogomphus* (47 species), *Chloropetalia* (4 species), and *Watanabeopetalia* (4 species) [1]. Although *C. papilio* has been studied by Karube Haruki, it has not been placed in any subgenus [11], and its phylogenetic relationship remains unresolved.

Mitochondria are important organelles in eukaryotic cells [12]. As semi-autonomous organelles, mitochondria retain their genetic material—the mitochondrial genome [13,14]. Unlike single mitochondrial genes, the complete mitochondrial genome provides more comprehensive genetic data, making it widely used in molecular evolution and phylogeny studies [15]. Mitochondrial DNA is typically maternally inherited, with distinct strategies employed across species to ensure this mode of transmission [16,17]. The mitochondrial genome is characterized by rapid evolution, maternal inheritance, small size, conserved gene content, and relatively easy acquisition, making it crucial for evolutionary research [18,19].

With the advent of high-throughput sequencing technology, it has become increasingly convenient to obtain data on the mitochondrial genome sequence [20]. As a result, the number of studies on the mitochondrial genomes in various insect groups has steadily increased [21]. The insect mitochondrial genome is approximately 16 kb in length and contains 37 genes: 13 genes encoding energy metabolism-related proteins, 22 transfer RNA (tRNA) genes, and 2 ribosomal RNA (rRNA) genes. The spacing between genes is minimal, and gene lengths are relatively conserved [22]. In addition, non-coding regions, also known as control regions or AT-enriched regions [23], are critical regulatory regions for the replication and transcription of the mitochondrial genome [24]. The length of these regions varies significantly among insect species: *Drosophila melanogaster* has an AT-rich region of 4601 bp [25], *Blattella bisignata* has 1705 bp [26], *Tetrix japonica* has 531 bp [27], and *Ruspolia dubia* has only 70 bp [28].

There are three genera and fifty-five species of Chlorogomphidae known worldwide [1,29]; however, as of April 7, 2025, only one complete mitochondrial genome (*Chlorogomphus shanicus*, GenBank: OP572413.1) has been available from NCBI. No other complete mitochondrial genome of Chlorogomphidae has been reported. In this study, we used Illumina and Sanger sequencing techniques to study the mitochondrial genome of *C*. *papilio*, collected from the Huangshan Scenic Area in Anhui Province. We report the complete mitochondrial genome sequence of *C. papilio* and analyze the evolutionary relationships among selected Chlorogomphidae species.

## 2. Materials and Methods

### 2.1. Test Materials

The two *C. papilio* specimens used in this experiment were collected by the author from the Huangshan Scenic Area, Anhui Province, China (30°09′12″ N, 118°14′32″ E) on 2 July 2022. The specimens were preserved in absolute ethanol at −20 °C for later use.

### 2.2. Genomic DNA Extraction and High-Throughput Sequencing

A single *C. papilio* individual was rinsed twice in ultrapure water, and the total DNA was extracted from the thoracic muscle tissue using the E.Z.N.A.^®^ Tissue DNA Kit (OMEGA, Beijing, China) according to the manufacturer’s instructions [30]. DNA concentration and purity were assessed using a Qubit 3.0 Fluorometer and 1% agarose gel electrophoresis. Subsequently, 1 μg of qualified DNA (purity: OD260/280 = 1.8–2.0; total amount ≥ 10 μg; concentration ≥ 50 ng/μL) was fragmented to 300–500 bp. The whole-genome library was prepared using the Illumina TruSeq™ Nano DNA Kit (Illumina, San Diego, CA, USA), which involved end repair, A-tailing, and adapter ligation, followed by 8-cycle PCR amplification in a 50 μL reaction volume (30–40 μL DNA fragments, 5 μL primer mix, 25 μL 2× master mix, and nuclease-free water to adjust the final volume to 50 μL). PCR conditions were as follows: initial denaturation at 98 °C for 30 s; 8 cycles of denaturation at 98 °C for 10 s, annealing at 60 °C for 30 s, and extension at 72 °C for 30 s; followed by a final extension at 72 °C for 5 min.

PCR products were size-selected (300–500 bp) by 2% agarose gel electrophoresis, purified, and subjected to paired-end sequencing (2×150 bp) with a sequencing depth of 22× on an Illumina NovaSeq 6000 platform at Beijing Qingke Biotechnology Co., Ltd. (Beijing, China).

### 2.3. Mitochondrial Genome Assembly and Annotation

Raw reads were processed with Trimmomatic v0.39 (http://www.usadellab.org/cms/index.php?page=trimmomatic accessed on 4 May 2024) [31] under default parameters for quality control. This included removal of adapter contamination, elimination of non-AGCT bases at the 5′ end, trimming of low-quality bases (Q < 20) at read ends, discarding reads with >10% N content, and exclusion of fragments < 75 bp post-trimming. The resulting dataset consisted of high-quality reads suitable for downstream analyses. The PacBio Sequel II platform sequencing data in BAM format were converted to FASTQ format through a rigorous quality control pipeline. This process involved the following: (1) initial length-based filtration to remove polymerase reads shorter than 200 bp, (2) quality filtering to eliminate reads with quality scores below 0.80, (3) extraction of subreads followed by adapter trimming, and (4) a final length-based filtration step to exclude any remaining subreads shorter than 200 bp. This multi-stage preprocessing protocol produced high-fidelity PacBio third-generation sequencing data, suitable for downstream genomic analyses.

The Illumina sequencing data were assembled using GetOrganelle v1.7.5 (https://github.com/Kinggerm/GetOrganelle accessed on 6 May 2024) [32]. BWA v0.7.17 [33] was used to align the second-generation assembly sequence with the third-generation data of PacBio [34], and the third-generation data of *C. papilio* were extracted. The extracted third-generation data were mixed with second-generation data using SPAdes v3.14.1 [35], and a sequence with sufficient coverage depth and long assembly length was selected as the candidate sequence. The mitochondrial scaffold sequence was confirmed by comparison with the NT library [36], and the sequences were connected according to the overlaps. Clean reads were aligned to the mitochondrial genome sequence, and the bases were corrected using Pilon v1.23 [37]. Finally, the starting position and direction of the mitochondrial assembly sequence were determined based on the reference genome, and the final mitochondrial genome sequence was obtained. The complete mitochondrial genome sequence was uploaded to the MITOS Web Server (http://mitos.bioinf.uni-leipzig.de/index.py accessed on 6 May 2024) [38] for gene function annotation to obtain initial annotation results of the mitochondrial genome. Redundancy in the initial gene predicted by MITOS was removed, and the start and stop codon positions of the gene were manually corrected to obtain a highly accurate conserved gene set. CGView software (http://stothard.afns.ualberta.ca/cgview_server/ accessed on 6 May 2024) [39] was used to display the *C. papilio* genome. Annotation of predicted genes was conducted via BLAST [40] analysis against the nucleotide database of NCBI (nt library) using BLAST 2.2.30+ (e-value < 1 × 10^−5^) based on the sequencing depth of the assembled genome. The annotated sequences were uploaded to the NCBI GenBank database to obtain the accession number (GenBank: PV287725).

### 2.4. Analysis of Mitochondrial Genome Characteristics

This study systematically analyzed the characteristics of the mitochondrial genome of *C. papilio*, including base composition, codon usage, and amino acid usage. MEGA v11.0 was used to calculate the nucleotide base composition, including the A + T content, AT-skew, and GC-skew. The skew values were computed using the following formulas: AT-skew = (A − T)/(A + T) and GC-skew = (G − C)/(G + C) [41]. Amino acid usage and relative synonymous codon usage (RSCU) of PGCs were analyzed using the codonW v1.4.4 software (http://codonW.sourceforge.net accessed on 7 May 2024) [42]. The secondary structure of the tRNA genes in the mitochondrial genome of *C. papilio* was predicted using the MITOS Web Server (http://rna.urmc.rochester.edu/RNAstructureWeb/ accessed on 7 May 2024) [38], and the secondary structure was visualized using RNAplot from the ViennaRNA package (v2.5.1) [43].

### 2.5. Phylogenetic Analyses

In this study, we conducted comprehensive phylogenetic analyses using 24 species from Chlorogomphidae and newly obtained species *C. papilio*, with six species from Cordulegastridae used as outgroups to construct a phylogenetic tree (Table 1). Nucleotide sequences of their mitochondrial *16S rRNA* and *cox1* genes (from the NCBI database) were used to construct the dataset. Subsequently, we used MEGA v11.0 [44] in conjunction with MUSCLE v3.8 [45] with default parameters to align the nucleotide sequences of the *16S rRNA* and *cox1* genes from 31 mitochondrial genomes, followed by manual trimming and alignment. PhyloSuite software was used to connect the two gene sequences after comparison. ModelFinder [46] was used to evaluate the optimal model of gene sequence.

Bayesian inference (BI) was performed using MrBayes v3.2 [47], with the GTR + I + G model selected based on ModelFinder [46]. The analysis was run for 10 million generations, with 25% [48] of the initial samples discarded as burn-in, and a phylogenetic tree was constructed. Maximum likelihood (ML) and maximum parsimony (MP) analyses were conducted using MEGA v11.0 [44] under the same model, with 1000 bootstrap replicates to assess branch support [49].

**Table 1:** Mitochondrial genomic information used in phylogenetic analyses of Chlorogomphidae in this study.

Species	GenBank Sequence Number		Reference
	*16S rRNA*	*cox1*	
*Chlorogomphus aritai*	LC366454.1	LC366751.1	Futahashi, 2014 [50]
*Chlorogomphus arooni*	LC366488.1	LC366785.1	Futahashi, 2014 [50]
*Chlorogomphus auratus*	LC366510.1	LC366807.1	Futahashi, 2014 [50]
*Chlorogomphus brevistigma*	LC200915.1	LC200926.1	Futahashi, 2014 [50]
*Chlorogomphus brunneus*	LC366602.1	LC366899.1	Futahashi, 2014 [50]
*Chlorogomphus caloptera*	LC366445.1	LC366742.1	Futahashi, 2014 [50]
*Chlorogomphus hiten*	LC366512.1	LC366809.1	Futahashi, 2014 [50]
*Chlorogomphus iriomotensis*	LC200916.1	LC200927.1	Futahashi, 2014 [50]
*Chlorogomphus kitawakii*	LC366444.1	LC366741.1	Futahashi, 2014 [50]
*Chlorogomphus magnificus*	LC366469.1	LC366766.1	Futahashi, 2014 [50]
*Chlorogomphus miyashitai*	LC366385.1	LC366682.1	Futahashi, 2014 [50]
*Chlorogomphus nakamurai*	LC366441.1	LC366738.1	Futahashi, 2014 [50]
*Chlorogomphus nasutus*	LC366459.1	LC366756.1	Futahashi, 2014 [50]
*Chlorogomphus okinawensis*	LC200917.1	LC200928.1	Futahashi, 2014 [50]
*Chlorogomphus piaoacensis*	LC366386.1	LC366683.1	Futahashi, 2014 [50]
*Chlorogomphus risi*	LC200919.1	LC200930.1	Futahashi, 2014 [50]
*Chlorogomphus shanicus*	LC366451.1	LC366748.1	Futahashi, 2014 [50]
*Chlorogomphus suzukii*	LC366344.1	LC366641.1	Futahashi, 2014 [50]
*Chlorogomphus tunti*	LC366453.1	LC366750.1	Futahashi, 2014 [50]
*Chlorogomphus vietnamensis*	LC366468.1	LC366765.1	Futahashi, 2014 [50]
*Chlorogomphus yokoii*	LC200921.1	LC200932.1	Futahashi, 2014 [50]
*Chloropetalia owadai*	LC366460.1	LC366757.1	Futahashi, 2014 [50]
*Watanabeopetalia uenoi*	LC366456.1	LC366753.1	Futahashi, 2014 [50]
*Watanabeopetalia usignata*	LC366458.1	LC366755.1	Futahashi, 2014 [50]
*Anotogaster klossi*	AB707862.1	AB708806.1	Futahashi, 2011 [51]
*Anotogaster kuchenbeiseri*	AB707879.1	AB708823.1	Futahashi, 2011 [51]
*Anotogaster chaoi*	AB707859.1	AB708803.1	Futahashi, 2011 [51]
*Anotogaster gregoryi*	LC366511.1	LC366808.1	Futahashi, 2014 [50]
*Anotogaster sakaii*	LC366345.1	LC366642.1	Futahashi, 2014 [50]
*Neallogaster pekinensis*	AB707904.1	AB708848.1	Futahashi, 2011 [51]

### 2.6. Divergence Time Estimation

We estimated divergence time using BEAST v2.6.2 [52], with the mitochondrial *cox1* gene as the molecular marker, employing both fossil calibration and molecular clock approaches. For the molecular clock method, we implemented an uncorrelated relaxed clock model [53], with a fixed evolutionary rate of 2.14% substitutions per site per million years (D/m.y.) for *cox1* [54]. For fossil calibration, we used the common ancestor of Cordulegastridae (sister group to Chlorogomphidae), constrained to 148.5 ± 3.6 Ma under a normal distribution prior [8]. We selected the normal distribution and uncorrelated relaxed clock model as the prior distributions. The analysis was conducted with the following settings: data partitioning and substitution models were selected based on ModelFinder results; the MCMC chain ran for 50 million generations, with samples taken every 1000 generations [54]. Convergence was assessed in Tracer v1.7 [55], ensuring that effective sample sizes (ESS) exceeded 200 for all parameters. Finally, TreeAnnotator v1.8.3 [56] (BEAST v2.6.2) was used to discard the top 10% of trees (Burn-in = 10%), select the Maximum Clade Credibility Tree, and calculate node heights using the average height values.

## 3. Results

### 3.1. Mitochondrial Genome Structure of C. papilio

**Figure 1:**
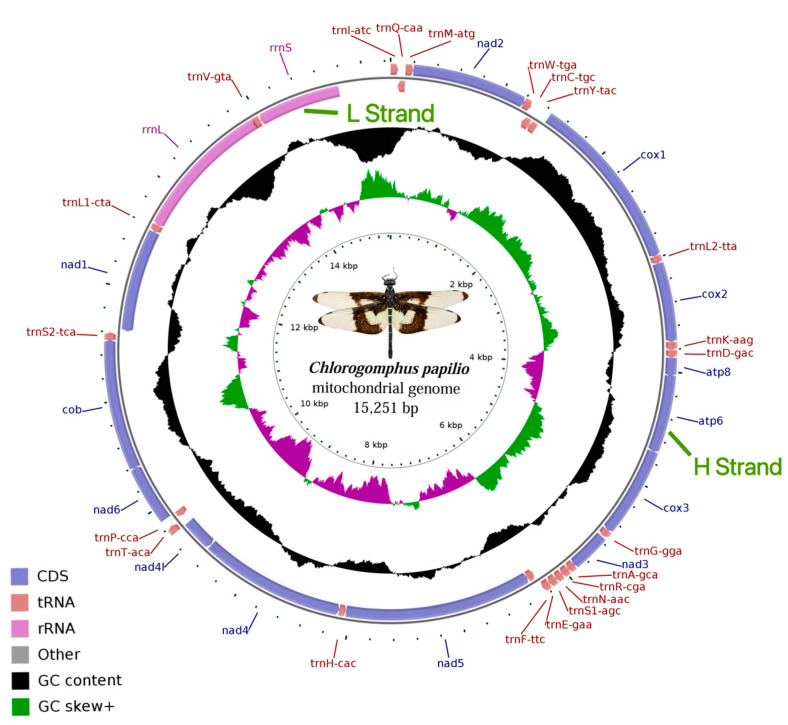
Mitochondrial genome structure of *Chlorogomphus papilio*. PCGs are represented by blue arrows, tRNA genes are depicted with brown arrows, and rRNA genes are shown using lavender arrows. tRNAs are labeled with single-letter amino acid codes, followed by their corresponding anticodons. Peaks on the black circular plot indicate the GC content, where outward and inward directions represent GC content above or below the average level, respectively. The purple and green circular plots illustrate the GC skew, with skew values between 0 and 1 shown in purple, and values between −1 and 0 shown in green. The ticks within the inner circle represent the sequence length.

The complete mitochondrial genome of *C. papilio* is 15,251 bp in length. It is a typical circular double-stranded DNA molecule containing 37 genes, including 13 PCGs, 22 tRNA genes, 2 rRNA genes (*12S rRNA* and *16S rRNA*), and 1 A + T-rich region. Of these, nine (*nad2*, *cox1*, *cox2*, *atp8*, *atp6*, *cox3*, *nad3*, *nad6*, *cob*), fourteen tRNA genes (*trnI*, *trnM*, *trnW*, *trnL*, *trnK*, *trnD*, *trnG*, *trnA*, *trnR*, *trnN*, *trnS1*, *trnE*, *trnT*, *trnS2*), and the control region are encoded on the majority (heavy, H) strand, also known as the J strand. Eight tRNA genes (*trnQ, trnC, trnY, trnF, trnH, trnP, trnL1, trnV*), four PCGs (*nad1*, *nad5*, *nad4*, *nad4L*), and two rRNA genes are encoded on the minority (light, L) strand, also referred to as the N strand (Figure 1; Table 2). As shown in Figure 1, the thickening of the inner circle indicates that the direction of gene transcription is from right to left, and the thickening of the outer circle indicates that the direction of gene transcription is from left to right.

### 3.2. Mitochondrial Genome Nucleotide Composition

The A + T content of the whole genome was 70.14% (A 38.95%, T 31.18%, G 11.82%, and C 18.04%). Transfer RNA genes had an A + T content of 71.28%, while ribosomal RNA genes showed 72.87%. The control region was particularly A + T-rich at 85.54%. Among PCGs, A + T content varied from 62% in *cox1* to 75.17% in *nad4l*.

The skew statistics of the entire nucleotide chain of *C. papilio* (AT-skew = −0.115, GC-skew = 0.04713) revealed a significant AT-skew and a moderate C-skew. Except for *nad5* (GC-skew = 0.313), *nad4* (GC-skew = 0.323), *nad4l* (GC-skew = 0.425), and *nad1* (GC-skew = 0.260), the GC-skew of the remaining nine PCGs was negative (−0.480 to −0.069). Except for *cox2* (AT-skew = 0.106), *atp8* (AT-skew = 0.046), *atp6* (AT-skew = 0), *cox3* (AT-skew = 0), *nad3* (AT-skew = 0.017), and *nad6* (AT-skew = 0.008), the AT-skew of the other seven genes was also negative (−0.403 to −0.025), indicating that the percentage of T and C contained in the 13 PCGs (54.79%) was higher than that of A and G (45.21%) (Table 3).

### 3.3. PCGs and Codon Usage

Among the 13 PCGs in the *C. papilio* mitochondrial genome, four genes were encoded on the minority strand (N), and the remaining nine genes are encoded on the majority strand (J) (Figure 1). All 13 PCGs used typical ATN start codons: ATA (3 genes), ATT (3), ATG (5), and ATC (2). Nine PCGs terminated with complete stop codons—TAA (seven genes) or TAG (2)—whereas *cox1*, *cox2*, *cox3*, and *nad5* used an incomplete stop codon (T) (Table 2).

**Figure 2:**
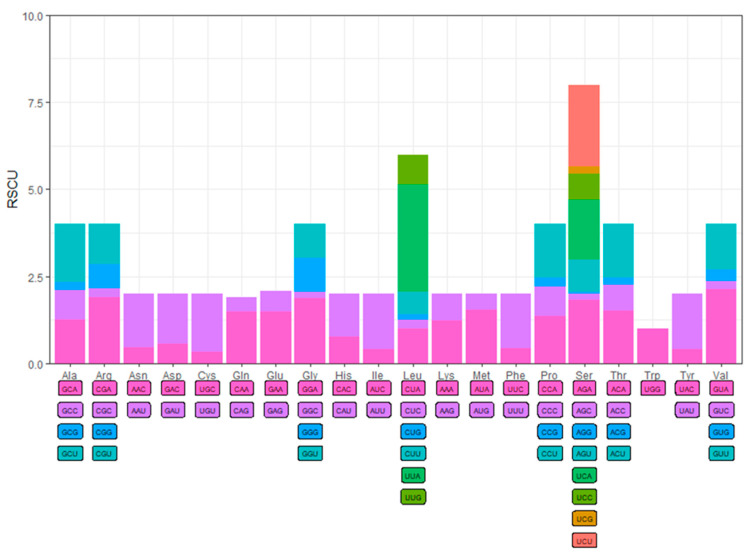
Relative synonymous codon usage (RSCU) of the mitogenomes of *Chlorogomphus papilio*.

The RSCU is a reference value for evaluating the frequency of codons encoding the same amino acid. The highest RSCU was UUA (F = 7.34%, RSCU = 3.081), and the lowest were AGG (F = 0.06%, RSCU = 0.046), CUG (F = 0.36%, RSCU = 0.151), and GGC (F = 0.30%, RSCU = 0.184). The top five codons with the highest frequency are AUU, UUA, UUU, AUA, and UAU, all of which are composed of A and U. The most frequently encoded amino acid in proteins was leucine (Leu), accounting for about 14.30%, followed by serine (Ser), isoleucine (Ile), phenylalanine (Phe), and methionine (Met) (Figure 2).

### 3.4. tRNA and rRNA

**Figure 3:**
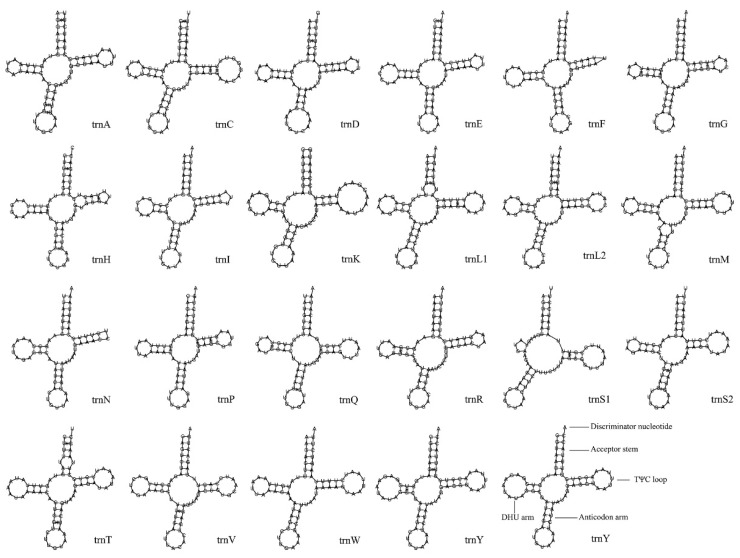
The secondary structure predictions of tRNA genes of *Chlorogomphus papilio*. The standard base pairing bonds of A and T, and C and G were represented by “-”, and the pairing bonds between G and U were indicated by “*”.

The mitochondrial genome of *C. papilio* contains 22 tRNA genes with a total length of 1490 bp. The longest genes are *trnW*, *trnC*, *trnY*, and *trnV* (71 bp), while the shortest genes were *trnI*, *trnG*, *trnA*, *trnF*, and *trnP* (65 bp). Among the 22 tRNA genes, 8 are encoded on the minority strand (N), and 14 are encoded on the majority strand (J) (Table 2, Figure 1). Except for three genes—*trnS1*, which lacks the DHU arm; *trnF*, which has an abnormal T-loop motif; and *trnN*, which lacks the TφC loop—all tRNA genes exhibit a typical cloverleaf secondary structure. In these structures, the amino acid acceptor arm generally forms 7 bp, except in *trnL1* and *trnT*, which have 6 bp. The anticodon arm forms 4 or 5 bp (4 bp in *trnD*, *trnK*, *trnM*, *trnR*), and the anticodon loop is consistently 7 bp. The TΨC arm ranges from 4 to 6 bp, and its loop varies from 1 to 11 bp. The DHU arm ranges from 3 to 4 bp, with a DHU loop length of 3 to 9 bp. The amino acid acceptor arms of *trnL1* and *trnT* each have an independent nucleotide, as does the TΨC arm of *trnH*. A total of 32 G–U base mismatches were identified in the tRNA secondary structures. These mismatches occurred in the DHU arms of *trnG* (2), *trnH*, *trnP*, *trnQ*, *trnR*, and *trnY* (7 total); in the amino acid acceptor arms of *trnA*, *trnC*, *trnD*, *trnE*, *trnH*, *trnL1*, *trnT*, and *trnY* (8 total); in the anticodon arms of *trnA* (2), *trnH*, *trnQ*, *trnS2* (2), *trnT*, and *trnV* (8 total); and in the TΨC arms of *trnC* (2), *trnG*, *trnH*, *trnL1*, *trnP* (2), *trnQ*, and *trnS1* (9 total) (Figure 3). These mismatched bases can be restored to normal pairing by post-transcriptional editing [43].

Two rRNA genes are ribosome large subunit (*rrnL*) and ribosome small subunit (*rrnS*). The length of *rrnL* gene sequence is 1316 bp, with an A + T content of 74.39%. The length of *rrnS* gene sequence is 774 bp, with an A + T content of 70.29%, showing an obvious AT bias (Table 2 and Table 3).

### 3.5. Control Region, Non-Coding, and Overlapping Areas

The length of the control region of the mitochondrial genome of *C. papilio* is 477 bp, located between *rrnS* and *trnI*. The content of the A, T, C, and G bases is 30.8%, 38.81%, 14.48%, and 11.82%, respectively, and the A + T content is 69.61%, showing an obvious AT base bias (Table 2 and Table 3). The mitochondrial genome of C. papilio consists of 10 non-coding regions (also called intergenic spacer regions) with lengths ranging from 1 to 43 bp, the longest of which is 43 bp, located between *trnY* and *cox1*. Moreover, 13 gene overlaps were observed, ranging from 1 to 23 bp, the longest (23 bp) occurring between *rrnL* and *trnV* (Figure 1; Table 2).

### 3.6. Phylogenetic Relationships

We constructed the phylogenetic tree of *C. papilio* using three independent methods (Bayesian inference, maximum likelihood, and maximum parsimony), all of which produced identical tree topologies. The topology selected was based on the Bayesian method. *C*. *papilio* and *C*. *magnificus* were clustered into a separate branch, and these were sister groups (BPP = 100%, ML = 100%, MPBP = 100%). In addition, *Chloropetalia owadai* and *Watanabeopetalia uenoi* clustered together with strong support (BPP = 100%, ML = 100%, MPBP = 100%). These groups were sister groups to six species of *Orogomphus* (BPP = 81%, ML = 100%).

**Figure 4:**
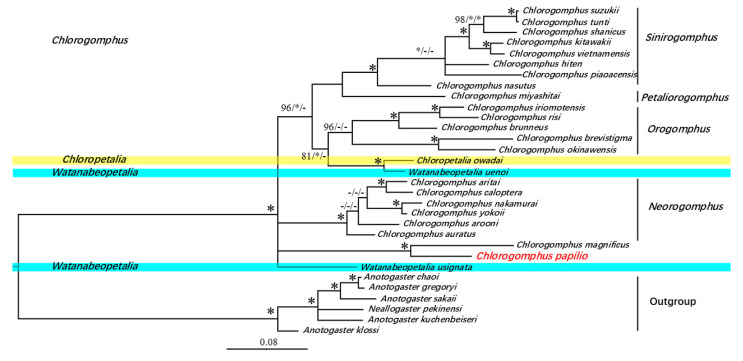
BI tree based on *16S rRNA* and *cox1* bigene data. All analysis support rates are marked near the node in BI tree/ML tree/MP tree order. The support rate within the range of 99–100% is represented by “*”, and the support rate of all results is 99–100% is represented by a larger “*”. The corresponding analysis does not support the use of “-” for this node, which is used to indicate low support values.

At the subgenus level, *Chlorogomphus* includes four subgenera: *Sinirogomphus*, *Petaliorogomphus*, *Orogomphus*, and *Neorogomphus*. Within this framework, *Chlorogomphus miyashitai* (*Petaliorogomphus*) and eight species of *Sinirogomphus* clustered together as sister groups (BPP = 98%, MPBP = 100%). *Sinirogomphus* and *Orogomphus* then formed a sister group relationship (BPP = 96%, ML = 100%), and *Neorogomphus* and (*Sinirogomphus* + *Orogomphus*) were sister groups (BPP = 100%, ML = 100%, MPBP = 100%) (Figure 4).

### 3.7. Divergence-Time Estimation Analysis

**Figure 5:**
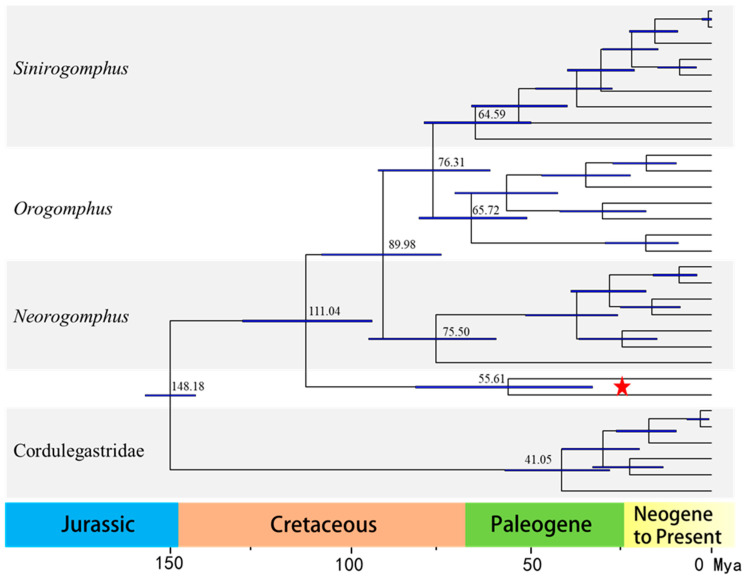
The divergence time estimations of family Chlorogomphidae. Blue bars indicate 95% mean confidence intervals of each node. A geological timescale is shown at the bottom. The red star indicates the specimen of *Chlorogomphus papilio*.

The estimated divergence time (Figure 5) suggests that the present-day Chlorogomphidae originated in the Early Cretaceous, approximately 111.04 Ma (95% confidence interval (CI): 92.91–128.47), slightly later than the divergence time of Aeschnidae (158–168 Ma). The common ancestor of *Sinorogomphus* diverged approximately 64.59 Ma (95% CI: 39.54–65.82), making it the earliest diverged subgenus of Chlorogomphidae (excluding the special case of *Watanabeopetalia*). *C. papilio* and *C*. *magnificus* diverged approximately 58.51 Ma (95% CI: 32.55–81.15 Ma). From Figure 5, it is evident that the subgenus differentiation in Chlorogomphidae occurred in the Late Cretaceous, while the family Chlorogomphidae itself emerged during the Paleogene.

## 4. Discussion

The mitochondrial genome of *C. papilio* is 15,251 bp in length, with the order and orientation of its 37 genes being consistent with those found in most insects [57,58,59]. No rearrangements were observed when compared to other published Chlorogomphidae mitochondrial genomes [60]. Additionally, the genome exhibits a distinct AT bias, a common feature among metazoans [57,61], with UUA showing the highest relative codon usage frequency. All 13 PCGs use standard ATN start codons, and four of them (*cox1*, *cox2*, *cox3*, *nad5*) terminate with an incomplete stop codon “T”, a common feature among arthropod mitochondrial genomes [62]. A similar situation occurs in other species of the Anax family. For example, *cox1*, *cox2*, and *nad5* of *Anax parthenope* end with T [58]. These incomplete stop codons are typically completed post-transcriptionally through polyadenylation, where the addition of a poly(A) tail generates functional stop codons [63,64,65]. Additionally, the analysis revealed a significant variation in codon usage, with a marked preference for codons containing A and U, particularly at the third position. This pattern is consistent with the strong AT bias commonly observed in mitochondrial genomes. A similar pattern has been widely reported in other arthropods [57], suggesting that this may be a conserved feature of mitochondrial genome evolution in this group. Leucine (Leu) was the most frequently encoded amino acid, followed by serine (Ser), isoleucine (Ile), phenylalanine (Phe), and methionine (Met), all of which also exhibit an AT composition. Among the 22 tRNA genes, most adopt the standard cloverleaf secondary structure, except *trnS1*, which lacks a DHU arm. A total of 32 G-U mismatches were detected in the secondary structures, a feature commonly found in arthropod tRNA [66]. A study on the mitochondrial tRNAs of spiders (Araneida) found that mismatched bases can be restored to normal pairing through post-transcriptional editing. The deletion of the DHU arm or T arm of the mitochondrial tRNA gene has little effect on normal function [67]. The two rRNA genes, separated by *trnV*, are located on the light strand and share the same characteristics as *C. shanicus* in the family Chlorogomphidae [60].

The results of the phylogenetic analyses showed that the topological structure of the phylogenetic tree constructed using the three methods was identical, which is consistent with traditional morphological classification [29]. In all analyses, *C. papilio* and *C. magnificus* formed a strongly supported sister group, representing a distinct and early-diverging lineage within Chlorogomphidae. These findings suggest that both species are among the most ancient lineages within the family. Although three genera of Chlorogomphidae were represented in the tree, the expected clear genetic divergence among them was not observed. *Watanabeopetalia usignata*, another species within *Watanabeopetalia* [68], appeared distantly related to both *W. uenoi* and *C. owadai*, indicating that additional data may be required to resolve the phylogenetic placement and genus-level classification of these three species. However, at the genus level, *C. owadai* was not separated from *Chlorogomphus*, and *W. uenoi* did not cluster with *W. usignata*. Due to limited genetic data for these genera, more extensive sampling is required to resolve their taxonomic positions.

Divergence time estimation analyses indicate that Chlorogomphidae originated in the Early Cretaceous, approximately 111.04 Ma, with subgenus differentiation occurring within the family during the Late Cretaceous. Fleck Ghunter proposed that the Libellulidae, a group closely related to Chlorogomphidae, can be traced back to the Late Cretaceous [69]. In accordance with this, Nel André suggested that *Araripechlorogomphus muratai*, the oldest known member of the family Chlorogomphidae, also dates back to the Late Cretaceous [70]. The family Chlorogomphidae emerged during the Paleogene, a period characterized by significant climatic shifts that likely influenced biodiversity. The rapid spread of flowering plants and grasses, along with the diversification of small insect prey such as Lepidoptera, Hymenoptera, and Diptera, provided essential ecological opportunities that facilitated the diversification and success of Chlorogomphidae. Among these, *C. papilio* gradually flourished during the Paleogene. *C. papilio* diverged approximately 58.51 Ma, a divergence likely associated with the Paleocene–Eocene Thermal Maximum (PETM), a period marked by rapid global warming as warm equatorial ocean currents mixed with cold Antarctic waters [71]. Simultaneously, tectonic activity—such as the separation of the Indian subcontinent from Africa and its subsequent collision with Asia—led to the uplift of the Himalayas [72]. These climatic and geological events created a warm, humid, and forested environment [73], which was ideal for insect diversification [74]. Coupled with geographic isolation resulting from the rising Himalayas, these environmental changes led to reproductive isolation in ancestral populations of *C. papilio*, driving its evolution into the distinct species we observe today. Due to the limited mitochondrial genome data available for Chlorogomphidae, the phylogenetic analyses in this study included only 25 Chlorogomphidae species and 6 Cordulegastridae species as outgroups. The sample size and species coverage were limited, which might not fully reflect the phylogenetic relationships of this family. Due to the current lack of complete mitochondrial genome data for many Chlorogomphidae species in public databases—with most species only represented by partial sequences such as *16S rRNA* and *cox1* genes—future studies should aim to expand the sampling scope by including a broader range of Chlorogomphidae species, especially those without full mitochondrial genome information, in order to provide a more comprehensive and accurate understanding of their phylogenetic relationships.

## 5. Conclusions

In this study, the complete mitochondrial genome of *C. papilio* was sequenced, assembled, and annotated for the first time. This represents the first report of the complete mitochondrial genome sequence for *C. papilio* and the second comprehensive mitochondrial genome analysis within the family Chlorogomphidae. A phylogenetic tree of 25 Chlorogomphidae species was constructed to provide a new perspective on the phylogenetic status and evolution of *C*. *papilio*. However, further phylogenetic studies incorporating additional genera and species within this family are needed to fully elucidate the molecular evolution and taxonomic relationships of Chlorogomphidae. Mitochondrial genome sequences are important resources for further molecular and phylogenetic analyses of Chlorogomphidae.

## Figures and Tables

**Table 2:** The 37 genes of the mitochondrial genome of *Chlorogomphus papilio*.

Gene	Strand	Start Position	Stop Position	Gene Length (bp)	Intergenic Nucleotide (bp)	Overlapping Nucleotide (bp)	Start Codon	Stop Codon
*trnI*	H	1	65	65	-	-	-	-
*trnQ*	L	63	130	68	-	3	-	-
*trnM*	H	130	198	69	-	1	-	-
*nad2*	H	199	1200	1002	-	-	ATT	TAA
*trnW*	H	1199	1269	71	-	2	-	-
*trnC*	L	1262	1332	71	-	8	-	-
*trnY*	L	1333	1403	71	-	-	-	-
*cox1*	H	1447	2983	1537	43	-	ATA	T
*trnL*	H	2984	3050	67	-	-	-	-
*cox2*	H	3051	3738	688	-	-	ATG	T
*trnK*	H	3739	3809	71	-	-	-	-
*trnD*	H	3810	3875	66	-	-	-	-
*atp8*	H	3876	4034	159	-	-	ATC	TAA
*atp6*	H	4031	4705	675	-	4	ATA	TAA
*cox3*	H	4705	5491	787	-	1	ATG	T
*trnG*	H	5492	5556	65	-	-	-	-
*nad3*	H	5566	5910	345	9	-	ATA	TAG
*trnA*	H	5909	5973	65	-	2	-	-
*trnR*	H	5974	6041	68	-	-	-	-
*trnN*	H	6042	6107	66	-	-	-	-
*trnS1*	H	6108	6175	68	-	-	-	-
*trnE*	H	6177	6244	68	1	-	-	-
*trnF*	L	6243	6307	65	-	2	-	-
*nad5*	L	6308	8036	1729	-	-	ATT	T
*trnH*	L	8037	8102	66	-	-	-	-
*nad4*	L	8102	9433	1332	-	1	ATT	TAG
*nad4l*	L	9439	9732	294	5	-	ATG	TAA
*trnT*	H	9735	9803	69	2	-	-	-
*trnP*	L	9814	9878	65	10	-	-	-
*nad6*	H	9880	10,398	519	1	-	ATC	TAA
*cob*	H	10,398	11,531	1134	-	1	ATG	TAA
*trnS2*	H	11,530	11,596	67	-	2	-	-
*nad1*	L	11,614	12,564	951	17	-	ATG	TAA
*trnL1*	L	12,567	12,634	68	2	-	-	-
*rrnL*	L	12,634	13,949	1316	-	1	-	-
*trnV*	L	13,927	13,997	71	-	23	-	-
*rrnS*	L	14,001	14,774	774	3	-	-	-

**Table 3:** Nucleotide composition of mitochondrial genome of *Chlorogomphus papilio*.

Regions	T (%)	C (%)	A (%)	G (%)	A + T (%)	AT Skew	GC Skew
Complete sequence	31.18	18.04	38.95	11.82	70.14	0.111	−0.208
tRNAs	35.03	12.35	36.24	16.38	71.28	0.017	0.14
rRNAs	39.43	10.1	33.44	17.03	72.87	−0.082	0.256
Control region	44.03	7.76	41.51	6.71	85.54	−0.029	−0.073
*atp6*	34.67	20	34.67	10.67	69.34	0	−0.304
*atp8*	32.7	23.27	35.85	8.18	68.55	0.046	−0.48
*cob*	34.39	19.75	31.48	14.37	65.87	−0.044	−0.158
*cox1*	32.08	20.95	29.93	17.05	62.01	−0.035	−0.103
*cox2*	29.36	19.48	36.34	14.83	65.7	0.106	−0.136
*cox3*	32.53	18.68	32.53	16.26	65.06	0	−0.069
*nad1*	48.05	10.94	22.4	18.61	70.45	−0.364	0.26
*nad2*	37.23	16.27	35.43	11.08	72.66	−0.025	−0.19
*nad3*	33.62	19.42	34.78	12.17	68.4	0.017	−0.23
*nad4*	48.5	9.68	22.9	18.92	71.4	−0.359	0.323
*nad4l*	52.72	7.14	22.45	17.69	75.17	−0.403	0.425
*nad5*	46.73	9.72	24.99	18.57	71.72	−0.303	0.313
*nad6*	36.61	16.76	37.19	9.44	73.8	0.008	−0.279
*rrnL*	39.59	9.35	34.8	16.26	74.39	−0.064	0.27
*rrnS*	39.15	11.37	31.14	18.35	70.29	−0.114	0.235
First position	38.5	13.83	31.42	16.25	69.92	−0.101	0.08
Second position	38.15	17.03	28.3	16.52	66.45	−0.148	−0.015
Third position	40.97	15.9	28.97	14.15	69.94	−0.172	−0.058

## Data Availability

The data presented in this study are available in NCBI GenBank (Accession number: PV287725).

## Abbreviations

The following abbreviations are used in this manuscript:

DOAJ	Directory of open access journals
TLA	Three letter acronym
LD	Linear dichroism
